# Screening of differentially expressed proteins from syncytiotrophoblast for severe early-onset preeclampsia in women with gestational diabetes mellitus using tandem mass tag quantitative proteomics

**DOI:** 10.1186/s12884-018-2066-9

**Published:** 2018-11-07

**Authors:** Xiaotong Sun, Tao Qu, Xiyan He, Xueping Yang, Nan Guo, Yan Mao, Xianghong Xu, Xiaodong Sun, Xuehong Zhang, Weihua Wang

**Affiliations:** 1 The First Clinical Medical College, Lanzhou University, Lanzhou, China; 2 Department of Obstetrics, Gansu Provincial Hospital, Lanzhou, China; 3 Department of Biotherapy Center, Gansu Provincial Hospital, Lanzhou, China; 4 Department of Endocrinology, Affiliated Hospital of Weifang Medical University, Weifang, China; 5 The Reproductive Medicine Special Hospital of the First Hospital of Lanzhou University, Lanzhou, China; 6 Key Laboratory for Reproductive Medicine and Embryo of Gansu, Lanzhou, China; 7 Houston Fertility Laboratory, Houston, TX USA

**Keywords:** Preeclampsia, Gestational diabetes mellitus, Syncytiotrophoblast, TMT technology, Biomarkers

## Abstract

**Background:**

Previous studies have revealed that women with gestational diabetes mellitus (GDM) have an increased risk of developing preeclampsia (PE). The possible reason is the abnormal lipid metabolism caused by GDM that leads to dysfunction of vascular endothelial cells and atherosclerosis, resulting in the onset of PE. However, studies focusing on the pathogenesis of PE in syncytiotrophoblast of GDM patients are lacking. This study aimed to compare differentially expressed proteins from syncytiotrophoblast between women with GDM and women with GDM with subsequently developed PE.

**Methods:**

Syncytiotrophoblast samples were obtained from pregnant women immediately after delivery. To explore the protein expression changes of syncytiotrophoblast that might explain the pathogenesis of PE in women with GDM, quantitative proteomics was performed using tandem mass tag (TMT) isobaric tags and liquid chromatography-tandem mass spectrometry. Bioinformatics analysis was performed to enrich the biological processes that these differentially expressed proteins were involved in.

**Results:**

A total of 28,234 unique peptides and 4140 proteins were identified in all samples. Among them, 23 differentially expressed proteins were identified between patients with GDM and patients with GDM with subsequently developed PE. Therein, 11 proteins were upregulated and 12 proteins were downregulated. Two relative proteins (FLT1 and PABPC4) were independently verified using immunoblotting analysis. Bioinformatic results indicated that the onset of PE in patients with GDM is a multifactorial disorder, involving factors such as apoptosis, transcriptional misregulation, oxidative stress, lipid metabolism, cell infiltration and migration, and angiogenesis.

**Conclusion:**

These results indicated that the inadequacy of endometrium infiltration, angiogenic disorder, and oxidative stress in syncytiotrophoblast are more likely to occur in patients with GDM and may be the potential mechanisms leading to such patients secondarily developing severe early-onset PE.

## Background

Preeclampsia (PE), which is one of the major causes of maternal and perinatal mortality or serious morbidity, is a common pregnancy-specific placental disease, affecting at least 5% of pregnant women [1–3]. PE is characterised by new-onset maternal hypertension and proteinuria after 20 weeks’ gestation [3, 4]. According to the onset time, PE can be divided into two subtypes: early-onset subtype, occurring before 34 weeks of gestation, and late-onset subtype, occurring from 34 weeks [5]. Although the known pathogenesis of PE is so diverse and complex that no hypothesis has so far covered all the causes, recent studies suggest that the early-onset and late-onset subtypes arise from different etiopathogenic causes. Early-onset PE is associated with abnormal placental function, mainly manifested in the insufficient invasion of the maternal myometrium by the trophoblast cells, which results in uterine spiral artery remodelling disorder and placental superficial implantation. Late-onset PE is associated with maternal predisposition to arterial diseases, developed due to interfering endothelial function by obesity, diabetes, lipid metabolism dysfunction, and inflammation [1, 6–8].

Gestational diabetes mellitus (GDM) also is a pregnancy-specific disease, characterised by the new onset of any degree of glucose intolerance during pregnancy [9]. Although GDM and PE share several similar risk factors such as increased pre-pregnancy body mass index (BMI) and maternal age as well as multiple gestation pregnancies, GDM was found to be an independent risk factor for PE [10, 11]. Women with GDM have an increased risk of developing PE [12–14]. One reason is that abnormal lipid metabolism caused by GDM leads to dysfunction of vascular endothelial cells and atherosclerosis [15]. This reason is consistent with the pathogenesis of late-onset PE.

Recently, high-throughput proteomic technology has been widely applied to screen differentially expressed proteins in different tissues of the placenta, plasma, syncytiotrophoblast extracellular vesicles, and umbilical artery from patients with PE [3, 16–19]. These studies partly revealed the potential pathogenesis of PE and identified candidate biomarkers to predict the occurrence of PE during pregnancy. Li et al. found that the level of plasma fatty acid-binding protein 4 (FABP4) in patients with PE in the GDM group was significantly higher than that in the GDM group, suggesting that plasma FABP4 level could predict the occurrence of PE in women with GDM [20]. However, no studies using high-throughput proteomic analysis to screen differentially expressed proteins between patients with GDM and patients with GDM with subsequently developed PE have been conducted. Therefore, this study aimed to establish a comparative proteomics of the syncytiotrophoblast in patients with GDM and patients with GDM with subsequently developed PE using the tandem mass tag (TMT) quantitative technology, in the hope of identifying candidate biomarkers for predicting PE from patients with GDM and providing targets for future therapy in women with PE with GDM.

## Methods

### Sample collection

This study was approved by the Ethical Committee of the Gansu Provincial Hospital. All study participants agreed to the sample collection and provided written informed consent. The inclusion criteria for all participants were the following: informed consent to participate in this study, age between 18 and 35 years, singleton pregnancy, absence of diabetes mellitus, hypertension, kidney disease, cardiovascular disease before pregnancy, and not taking any medication. A total of 24 patients with GDM were diagnosed according to a 75-g oral glucose tolerance test (OGTT) at 24–28 weeks of gestation (fasting ≥ 5.1 mmol/L, 2 h ≥ 8.5 mmol/L). All pregnant women with GDM were treated with diet without insulin intervention. Among them, 9 patients developed severe PE according to systolic blood pressure (SBP) ≥160 mmHg and/or diastolic blood pressure (DBP) ≥110 mmHg and proteinuria > 3 g/24 h and selected as cases.

Placental samples of the 9 patients with GDM and 9 patients with PE with GDM were obtained after the participants’ delivery at the Gansu Provincial Hospital. The syncytiotrophoblasts were separated from the placental samples within 30 min, frozen immediately in liquid nitrogen, and stored at − 80 °C.

### Protein extraction

Three samples from the 9 patients with GDM or 9 patients with PE with GDM were randomly selected as one biological parallel. An equal amount of syncytiotrophoblast sample from each patient was pooled and ground into powder in liquid nitrogen. All of the mixed samples were homogenised using homogeniser in SDT1 buffer (4% SDS, 1 mmol DTT, 100 mmol Tris–HCl, pH 7.6). Protein extraction was performed by sonication (Power 80 W, work 10 s, interval 10 s, cycle 10 times) on ice and then was boiled for 15 min. The crude extract was centrifuged at 14,000 g for 40 min to collect the supernatant. The supernatant was filtered with 0.22-μm filters and quantified with the BCA Protein Assay Kit (Bio-Rad, USA). The filtrates were stored at − 80 °C for future use.

### Protein digestion and TMT labelling

Protein digestion was performed using filter-aided sample preparation (FASP) procedure as described before [19]. Briefly, 200 μg of total proteins from each pool was diluted in 30 μl SDT2 buffer (4% SDS, 100 mmol DTT, 150 mmol Tris–HCl, pH 8.0), boiled for 5 min, and cooled to 25 °C. The proteins were washed using washing buffer (8 M urea, 150 mmol Tris–HCl, pH 8.0) by repeated ultrafiltration (Microcon units, 10 kD) at 14,000 g for 15 min to remove the low-molecular-weight components. Each filter was added in 100 μl iodoacetamide (100 mM iodoacetamide in washing buffer) for blocking reduced cysteine residues, incubated in darkness for 30 min, and then centrifuged to discard the filtrate. The filter was washed three times using 100 μl washing buffer and 100 μl of 100 mM TEAB buffer. Then, 4-μg trypsin (Promega, USA), revolved in 40-μl TEAB buffer, was added to digest the protein suspension overnight at 37 °C. Finally, the resulting peptides were collected via centrifugation, and the concentrations were calculated according to OD280.

About 100 μg of each resulting peptide mixture was labelled using TMT reagent according to the manufacturer’s instructions (Thermo Fisher Scientific, USA). Specifically, three syncytiotrophoblast samples from patients with GDM were labelled with 128 N, 128C, and 129 N isobaric TMT tags, while the other three syncytiotrophoblast samples from patients with PE with GDM were labelled with 129C, 130 N, and 130C isobaric TMT tags, respectively. After TMT labelling, the digested sample was fractionated into 10 fractions, and the excess label and salts were removed, according to Pierce High pH Reversed-Phase Fractionation Kit Manual (Thermo Fisher Scientific).

### Liquid chromatography–mass spectrometry/mass spectrometry analysis

Each fraction, dissolved into solution A (0.1% formic acid), was loaded into a reverse phase trap column (Thermo Scientific Acclaim PepMap100, 100 μm × 2 cm, nanoViper C18) connected to the C18-reversed phase analytical column (Thermo Scientific Easy Column, 10 cm long, 75-μm inner diameter, 3-μm resin) and separated with a linear gradient of solution B (84% acetonitrile and 0.1% formic acid) at a flow rate of 300 nL/min. The linear gradient was processed as follows: 0–55% solution B for 80 min, 55–100% solution B for 5 min, and 100% solution B for 5 min.

Ten fractions from each sample were analysed using liquid chromatography–mass spectrometry/ mass spectrometry (LC-MS/MS) by Q Exactive Mass Spectrometer coupled to Easy nLC (Thermo Scientific) in positive ion mode for 90 min. MS data was acquired using a data-dependent top10 method, dynamically choosing the most abundant precursor ions from the survey scan (300–1800 m/z) for higher-energy collisional dissociation (HCD) fragmentation. The instrument parameters were set as follows: automatic gain control target was 3e6; dynamic exclusion duration, 40 s; resolution for survey scans, 70,000 at m/z 200; resolution for HCD spectra, 35,000 at m/z 200; and isolation width, 2 m/z. Normalised collision energy was 30 eV, and the underfill ratio was 0.1%.

### Data analysis

The acquired MS spectra were analysed using MASCOT search engine (version 2.2; Matrix Science, London, UK) embedded into Proteome Discoverer 1.4 (Thermo Electron, San Jose, CA, USA). The parameters were set as follows: peptide mass tolerance was ±20 ppm, fragment mass tolerance was 0.1 Da, and max missed cleavages were set to 2. TMT-10plex was set as fixed modifications, and oxidation was set as variable modifications. False discovery rate of peptides was set to be < 0.01. All peptide ratios were normalised by the median protein ratio, and the median protein ratio was defined as 1 after the normalisation.

### Protein identification and bioinformatic analysis

For identification of differentially expressed proteins, the fold change should be > 1.2 or < 0.83 with a *P* value (student’s *t*-test) < 0.05. The gene ontology (GO) annotation of differentially expressed proteins was blasted against SwissProt database (human) using the NCBI BLAST**+** client software. Functional annotation was searched against the online Kyoto Encyclopedia of Genes and Genomes (KEGG) database (http://geneontology.org/). GO enrichment and KEGG pathway enrichment were performed based on the Fisher’ exact test. Hierarchical clustering was analysed using Cluster 3.0 (http://bonsai.hgc.jp/~mdehoon/software/cluster/software.htm) and the Java Treeview software. The protein–protein interaction (PPI) networks were analysed using IntAct Molecular Interaction Database (http://www.ebi.ac.uk/intact/) and visualised using Cytoscape software.

### Immunoblotting

For immunoblotting analysis, syncytiotrophoblast tissue was homogenised in RIPA buffer containing protease inhibitors and quantified with the BCA Protein Assay Kit. The protein bands were detected and imaged using a capillary-based western blot automated system ProteinSimple WES with anti-vascular endothelial growth factor receptor 1 (FLT1, 1:1000, Proteintech, 13,687–1-AP), anti-polyadenylate-binding protein 4 (PABPC4, 1:1000, Proteintech, 14,960–1-AP), and anti-glyceraldehyde-3-phosphate dehydrogenase (GAPDH, 1:1000, Proteintech, 10,494–1-AP).

## Results

### Characteristics of the patients

Table 1 shows the clinical characteristics of patients with GDM and patients with GDM with subsequently developed PE. The maternal age, parity, pregnancy BMI at 12 week, gestation age at delivery, gestation age at 75-g OGTT, and glucose concentrations of 0 and 2 h OGTT in patients with GDM did not differ significantly from those in patients with GDM with subsequently developed PE. Systolic and diastolic blood pressures, as well as insulin concentration, were significantly higher in patients with GDM with subsequently developed PE than that in patients with GDM (*P* < 0.05). The protein contents in a 24-h urine collection in all of the patients with GDM with subsequently developed PE were 5.56 ± 1.65 g, while those in all of the patients with GDM were 0.01 ± 0.01 g. It is worth noting that, without medication, all of the diagnoses of PE in patients with GDM in our study were severe and before 34 weeks (data not shown), suggesting that GDM promotes the development of severe early-onset PE.

**Table 1:** Clinical characteristics of patients with GDM and patients with GDM with subsequently developed PE (GDM/PE)

	GDM	GDM/PE	*P* value
Sample size (*n*)	9	9	
Maternal age (years)	32.67 ± 5.12	30.22 ± 3.90	0.107
Parity	0.44 ± 0.53	0.33 ± 0.47	0.681
Pregnancy BMI at 12 week (kg/m^2^)	25.76 ± 2.56	25.93 ± 2.14	0.824
Gestation age at delivery (wk)	38.49 ± 0.98	33.97 ± 1.05	< 0.001^*^
Gestation age at 75-g OGTT (wk)	24.70 ± 040	24.81 ± 0.49	1.100
OGTT 0 h glucose (mmol/L)	5.46 ± 0.64	5.31 ± 0.27	0.533
OGTT 2 h glucose (mmol/L)	7.36 ± 1.12	8.43 ± 1.39	0.027^*^
Insulin (mU/L)	11.73 ± 1.79	15.17 ± 3.09	0.046^*^
Maximum SBP (mmHg)	131.33 ± 5.70	186.44 ± 11.59	< 0.001^*^
Maximum DBP (mmHg)	85.22 ± 4.58	121.89 ± 9.46	< 0.001^*^
Proteinuria (g/24 h)	0.01 ± 0.01	5.56 ± 1.65	< 0.001^*^

^*^P < 0.05

### Protein identification

**Fig. 1:**
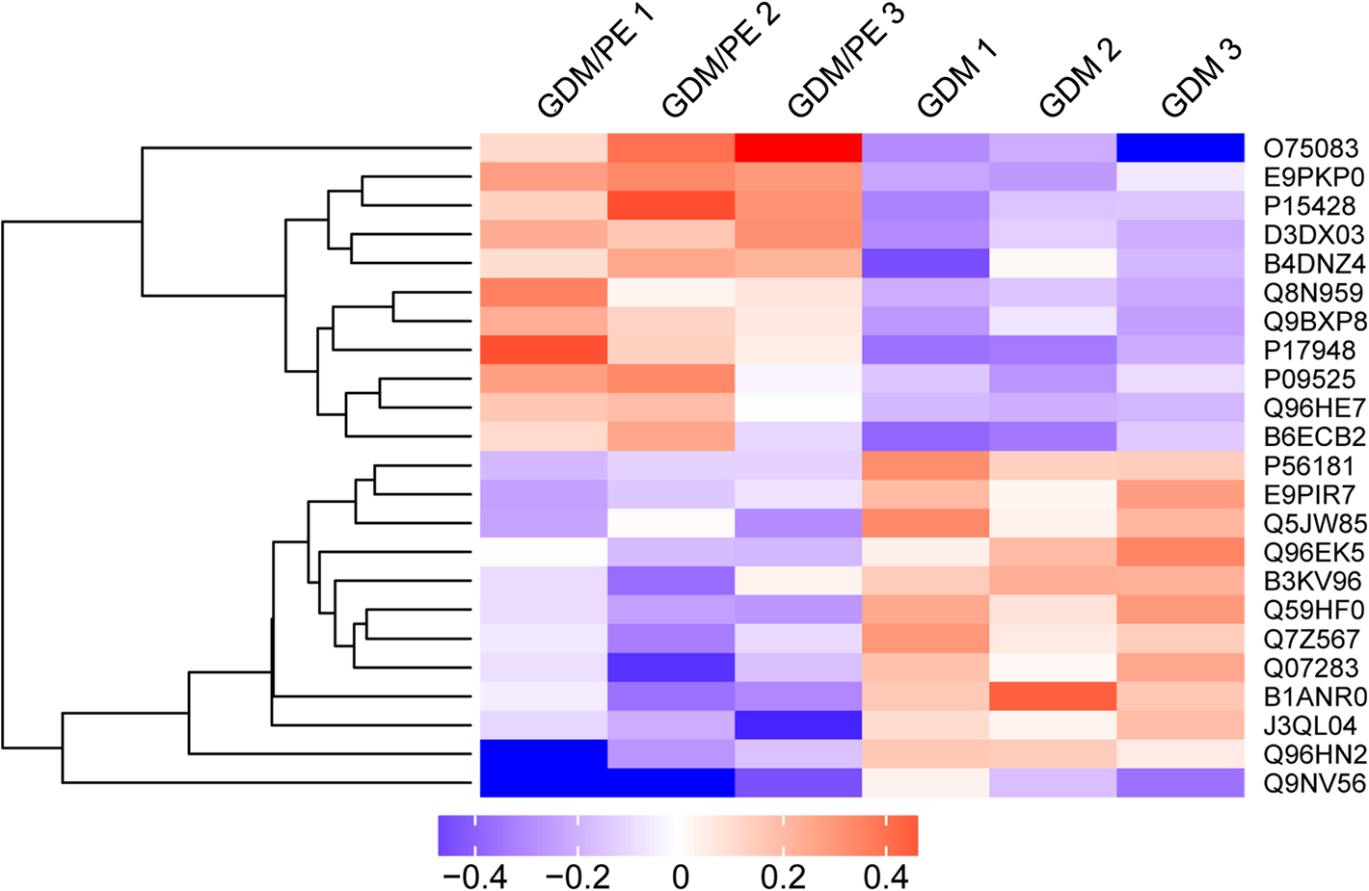
K-means clustering of differentially expressed proteins identified in human syncytiotrophoblast

A total of 28,234 unique peptides and 4140 proteins were identified in all samples. According to protein identification criteria, 23 differentially expressed proteins were found in comparison between the patients with PE with GDM and patients with GDM (Table 2). Among them, 11 proteins were upregulated, and 12 proteins were downregulated. The K-means clustering of these differentially expressed proteins is shown in a heat map (Fig. 1).

**Table 2:** The differentially expressed proteins identified in syncytiotrophoblast from patients with GDM with subsequently developed PE and patients with GDM

Protein name	Accession No	Gene name	Fold change	*P* value
WD repeat-containing protein 1	O75083	WDR1	1.600	0.026
Vascular endothelial growth factor receptor 1	P17948	FLT1	1.377	0.031
15-hydroxyprostaglandin dehydrogenase	P15428	HPGD	1.366	0.015
Ribonuclease H2, subunit C	E9PKP0	RNASEH2C	1.339	0.001
Amine oxidase	D3DX03	ABP1	1.311	0.003
cDNA FLJ51917	B4DNZ4	N/A	1.261	0.040
Mucin 1	B6ECB2	MUC1	1.256	0.049
Annexin A4	P09525	ANXA4	1.251	0.049
cDNA FLJ38330	Q8N959	N/A	1.235	0.045
Pappalysin-2	Q9BXP8	PAPPA2	1.221	0.015
ERO1-like protein 1 alpha	Q96HE7	ERO1A	1.205	0.011
KIF 1-binding protein	Q96EK5	KIF1BP	0.826	0.047
Activating transcription factor 3	Q7Z567	ATF3	0.823	0.041
Thioredoxin reductase 1	E9PIR7	TXNRD1	0.822	0.027
cDNA FLJ16285	B3KV96	N/A	0.815	0.035
NADH dehydrogenase flavoprotein 3	P56181	NDUFV3	0.815	0.010
Glutathione S-transferase A3	Q5JW85	GSTA3	0.802	0.045
Tether containing UBX domain for GLUT4	J3QL04	ASPSCR1	0.795	0.037
Trichohyalin	Q07283	TCHH	0.789	0.044
Solute carrier family 13	Q59HF0	N/A	0.781	0.009
MRG/MORF4L-binding protein	Q9NV56	MRGBP	0.776	0.042
Adenosylhomocysteinase 2	Q96HN2	AHCYL2	0.746	0.028
Polyadenylate-binding protein 4	B1ANR0	PABPC4	0.744	0.025

### Immunoblotting verification

**Fig. 2:**
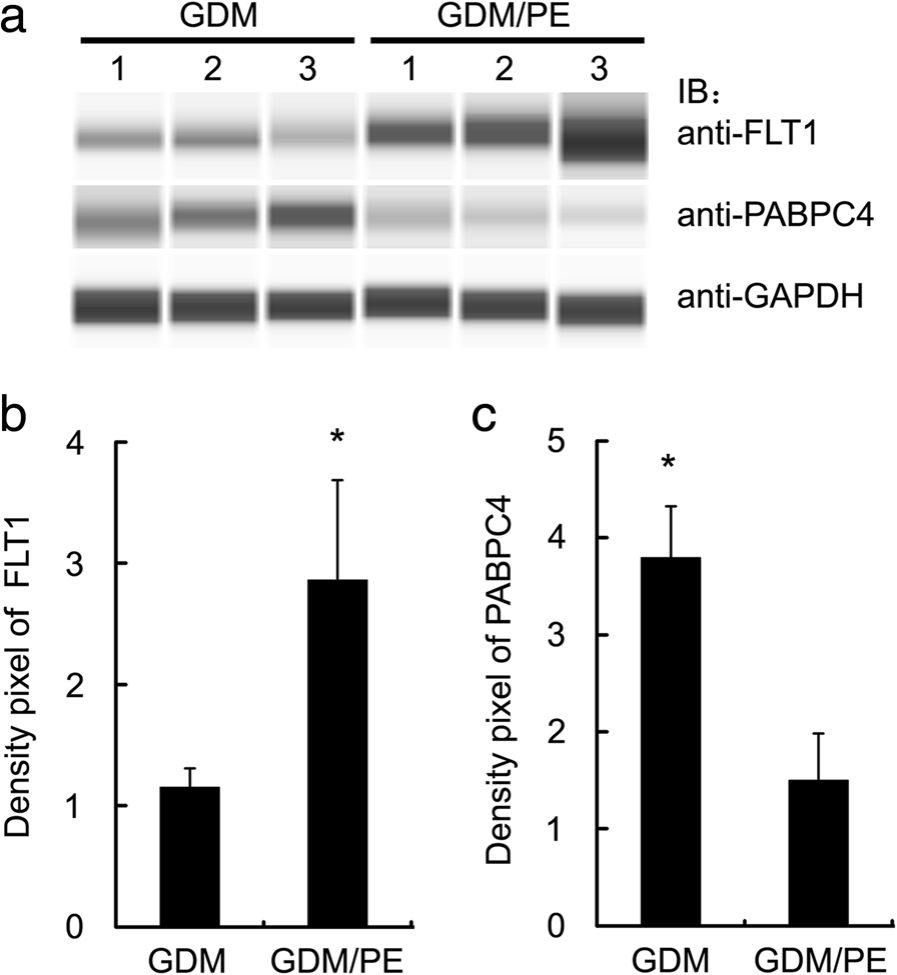
Immunoblotting analysis with anti-FLT1, anti-PABPC4 and anti-GAPDH antibodies was performed on patients with GDM and patients with GDM with subsequently developed PE (**a**). Comparison of protein expression levels of FLT1 (**b**) and PABPC4 (**c**) in patients with GDM with subsequently developed PE and patients with GDM groups. The Y-axis represents the relative quantification of target proteins normalised to GAPDH. The minimum mean of band density pixels was taken as 1

To verify the expression levels of differentially expressed proteins discovered by TMT analysis, immunoblotting was performed to investigate the expression patterns of FLT1 and PABPC4, two proteins in the same samples. GAPDH was chosen as an internal control. The results showed that FLT1 was expressed at higher levels, while PABPC4 was expressed at lower levels in PE with GDM groups than in GDM groups (Fig. 2), suggesting that the immunoblotting results were in line with TMT proteomics results.

### Bioinformatic analysis

**Fig. 3:**
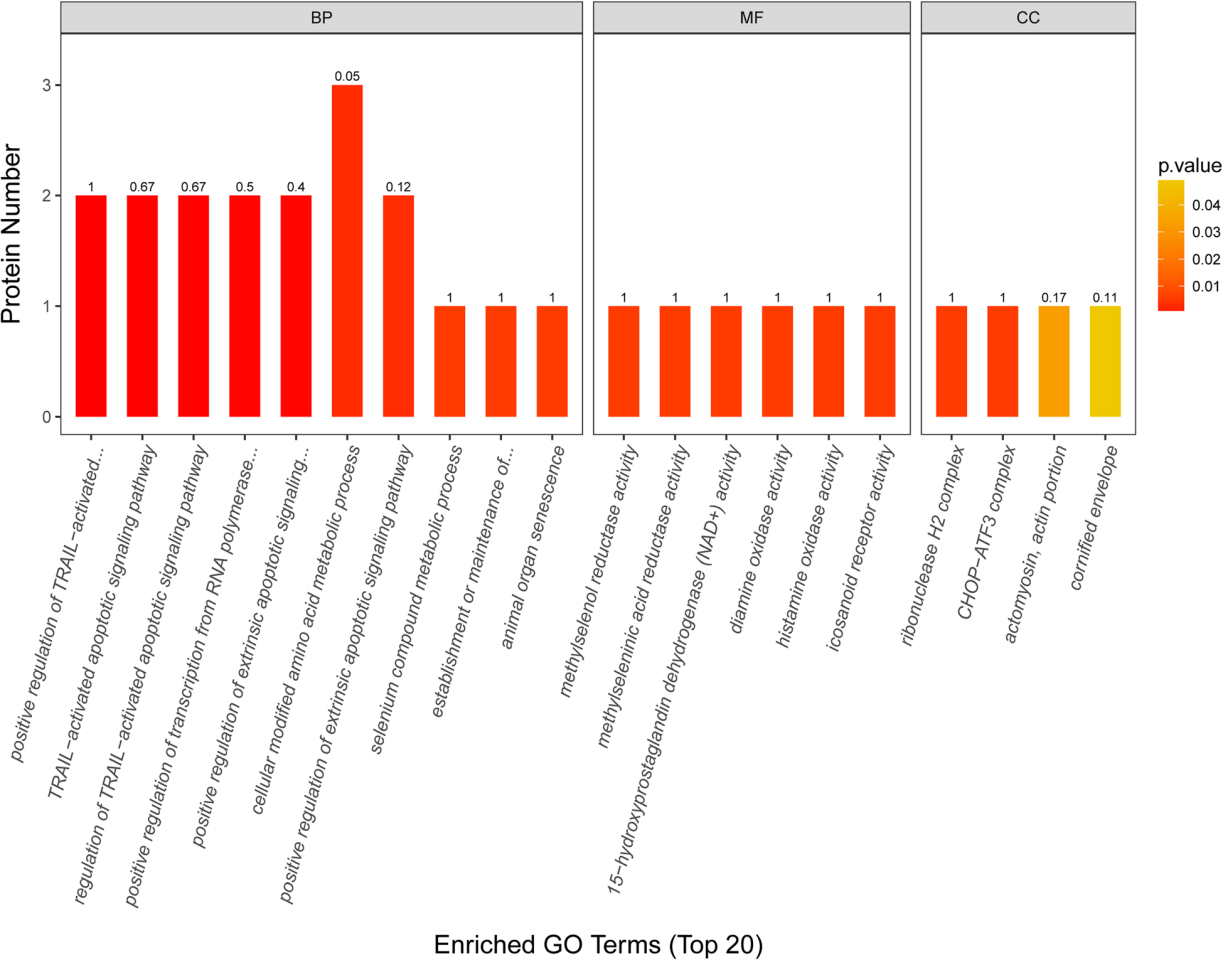
Bioinformatics analysis of the differentially expressed proteins. The top 20 rankings of biological process, molecular function, and cellular component that significantly changed based on GO analysis. The number on each bar represents the proportion of the differentially expressed proteins annotated to a GO category to the overall proteins annotated to the same GO category

**Fig. 4:**
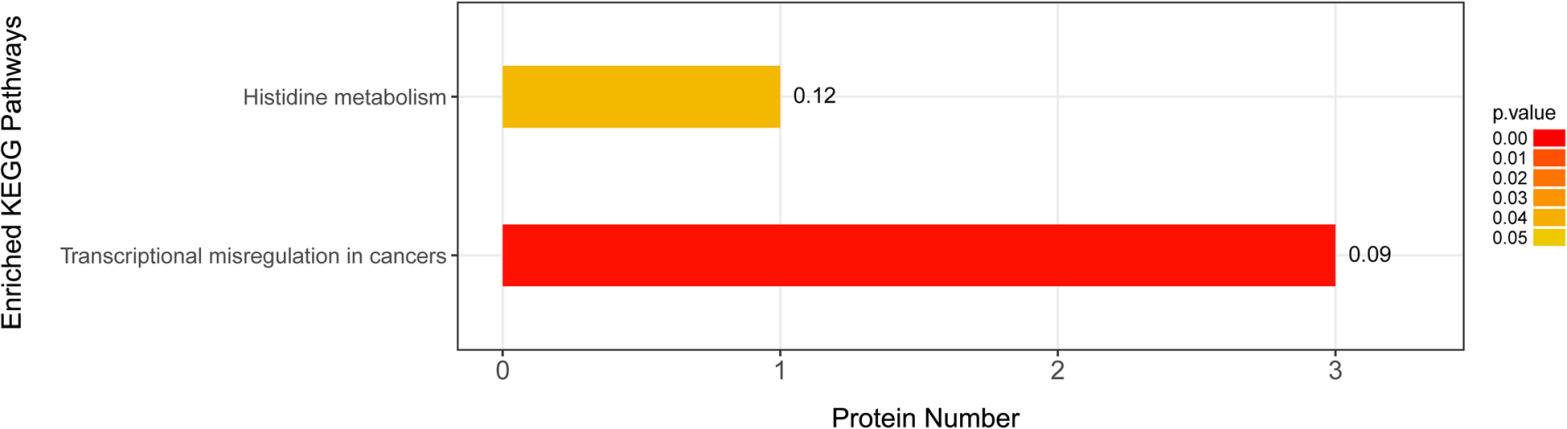
KEGG pathway enrichment of differentially expressed proteins. The number on each bar represents the proportion of differentially expressed proteins annotated to a KEGG pathway to the overall proteins annotated to the same KEGG pathway

**Fig. 5:**
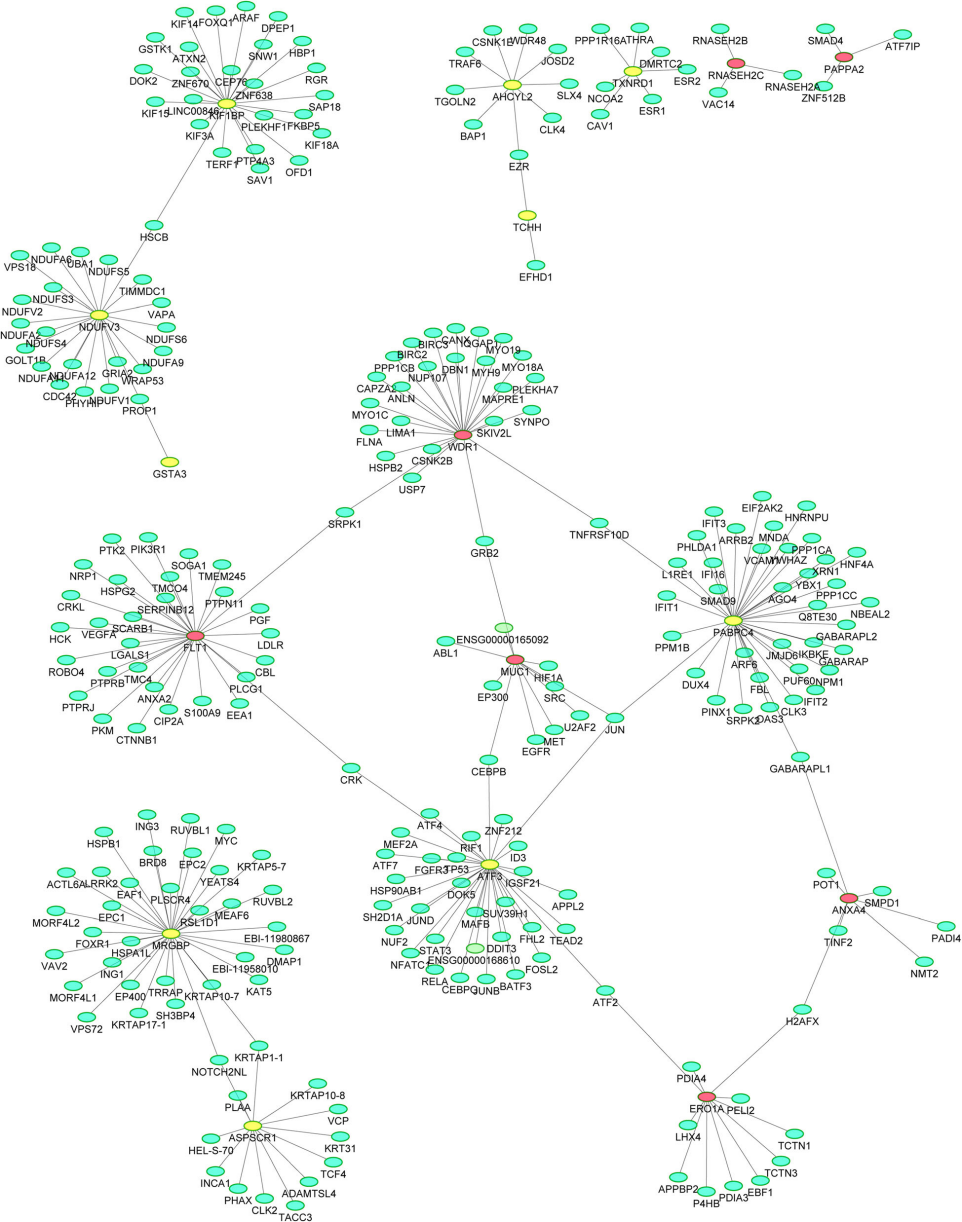
The protein interaction network of differentially expressed proteins. Red spot indicates upregulated protein, yellow spot indicates downregulated protein

Figure 3 shows the top 20 rankings of biological process, molecular function, and cellular component based on GO annotation. The main biological process of these differentially expressed proteins was regulation of extrinsic apoptotic signalling pathway, regulation of transcription from RNA polymerase, cellular modified amino acid metabolic process, selenium compound metabolic process, establishment or maintenance of polarity of follicular epithelium, and animal organ senescence. The main molecular function of these proteins was associated with methylselenol reductase activity, 15-hydroxyprostaglandin dehydrogenase activity, diamine oxidase activity, histamine oxidase activity, and icosanoid receptor activity. The main cellular component was ribonuclease H2 complex, CHOP–activating transcription factor 3 complex, actomyosin, actin portion, and cornified envelope. By KEGG pathway analysis, 4 differentially expressed proteins were enriched into histidine metabolism and transcriptional misregulation in cancer pathways (Fig. 4). By PPI analysis, 7 differentially expressed proteins (WDR1, FLT1, MUC1, ANXA4, ERO1A, ATF3, and PABPC4) interacted with each other and enriched into a larger protein interaction network. The other 10 differentially expressed proteins (KIF1BP, NDUFV3, and GSTA3; ASPSCR1 and MRGBP; AHCYL2 and TCHH; TXNRD1; RNASEH2C; and PAPP-A2) enriched into different protein interaction network, respectively (Fig. 5). These results suggested that occurrence of PE in women with GDM is the result of multiple pathway dysfunction.

## Discussion

Although studies have revealed that GDM is one of the risk factors for developing PE, the pathogenesis remains unclear. To clarify the molecular mechanisms and search the potential biomarkers for PE screening from women with GDM, we identified and quantified differentially expressed proteins from women with GDM with subsequently developed PE compared with GDM women. The results showed that a total of 23 proteins expressed differentially between the two groups. These proteins involved in different biological functions and signalling pathway implied that multiple mechanisms contribute to the development of PE from women with GDM.

Of the 23 differentially expressed proteins, 7 have been reported to be implicated in the onset of PE. In our study, 5 of these proteins have upregulated expression, such as FLT1, MUC1, ANXA4, PAPP-A2, and HPGD. Two proteins were downregulated, such as ATF3 and TXNRD1.

As a transcriptional repressor, ATF3 is induced rapidly when cells are exposed to a wide range of stress stimuli. The increased ATF3 prevents cells from tumour necrosis factor alpha (TNF-α)-induced apoptosis by inhibiting tumour suppressor gene p53 transcription and cleavage of procaspase 3. Thus, ATF3 may maintain the survival of endothelial cells during vascular inflammation and atherosclerosis [21]. In preeclamptic placentas, both mRNA and protein expression levels of ATF3 were decreased. This reduction was likely to occur after prolonged hypoxia. The decrease of ATF3 expression increased cytokine interleukin 6, TNF-α, and nuclear factor kappaB (NF-κB) expression as well as Flt-1 secretion [22]. FLT1 is produced in the placenta and can be detected in the placenta, amniotic fluid, and serum [23–25]. The function of FLT1 is to antagonise vascular endothelial growth factor (VEGF) effects on the formation of placental vasculature and maternal endothelial cell function [24]. The increase of FLT1 protein level is an important risk factor leading to impaired placental angiogenesis [26]. In fact, the expression level of FLT1 in serum of the large number of women with PE were significantly elevated [27–31], while after 48 h of delivery, the level of FLT1 in serum decreased dramatically [32, 33]. Patients with GDM tend to develop dysfunction of vascular endothelial cells and atherosclerosis, leading to PE [15]. Therefore, ATF3–FLT1 axis may be the primary pathogenesis of women with GDM to secondarily developed PE.

MUC1, with a large mucin-like extracellular domain, locates on the surface of cells. Overexpression of MUC1 reduces cell–cell interactions and inhibits integrin-mediated cell adhesion to the extracellular matrix [34]. Immunohistochemical and immunoblotting analysis showed that the expression levels of MUC1 in the syncytiotrophoblast and extravillous trophoblast cells (EVT) of severe preeclamptic placentas were increased. MUC1 overexpression suppressed cell–matrix adhesion and EVT invasion [35], probably leading to insufficient infiltration of syncytiotrophoblast cells into the endometrium.

ANXA4 is a Ca^2+^-dependent phospholipid binding protein, specifically expressing on the basal surface of syncytiotrophoblast. The increased activity of phospholipase A2 would lead to more arachidonic acid decomposing into thromboxane A2, which results in coagulation subsequently. Therefore, after delivery, ANXA4 enters the maternal bloodstream and prevent the activation of blood coagulation [36]. In gallbladder cancer tissues, the ANXA4 gene was highly expressed. Moreover, the elevated ANXA4 expression correlated well with invasion depth in patients with gallbladder cancer. One reason is that upregulated expression of ANXA4 promotes tumour cell growth, migration, and invasion by activating NF-κB pathway [37]. NF-κB plays important roles in the cell proliferation, inflammation, angiogenesis, apoptosis, invasion, and cellular adhesion, which also are important features in pathogenesis of PE. Using 2D electrophoresis combined with MALDI-TOF-MS, ANXA4 was identified as one of the 11 upregulated expressed proteins from preeclamptic placenta compared to that of normal pregnancy. This result is the first finding that there is a relationship between upregulated ANXA4 and the occurrence of PE [38]. In our study, ANXA4 also has upregulated expression in patients with GDM with subsequently developed PE compared to patients with GDM. The involved common mechanism should be further studied in detail.

Insulin-like growth factors (IGFs) and their binding proteins (IGFBPs) play important roles during establishment of pregnancy and placental development by mediating cell communication between trophoblasts and deciduae, as well as promoting trophoblast invasion [39]. PAPP-A2, also named pregnancy-associated plasma protein-A2, is capable of cleaving IGFBP5 specifically to regulate its biological function [40]. Both mRNA and protein expression levels of PAPP-A2 were upregulated in severe early-onset preeclamptic placentas and localised to the syncytiotrophoblast [41]. The upregulated PAPP-A2 may degrade IGFBP5 and then inhibit trophoblast invasion. However, a study has also shown that hypoxia and TNF-α, but not oxidative stress, contributed to the upregulation of PAPPA2, suggesting that PAPPA2 is upregulated as a consequence, rather than a cause of PE [42]. Due to the increased concentration of PAPP-A2 in serum in agreement with that in placenta, PAPP-A2 has a potential as a biomarker for the onset of PE [43].

Oxidative stress damages placental tissue resulting in intrauterine growth retardation and foetal distress, involving in the pathogenesis of PE. Thioredoxin reductase was located in the cytoplasm and mitochondria of cytotrophoblasts and deciduae in the placenta. It may protect the placenta against oxidative stress by reducing reactive oxygen species [44]. The decreased protein expression level of TXNRD1 may be attributed to patients with GDM with PE.

As a cancer suppressor, the expression level of HPGD is decreased in several tumour cells. The decreased HPGD blocks the production of the prostaglandin E2 (PGE2) metabolite, which can promote cancer progression by modulating tumour cells of proliferation, migration, invasion, angiogenesis, and apoptosis [45]. In a previous study, the mRNA level of HPGD in the placenta of patients with PE was decreased [46], while in our study, the protein level of HPGD was increased, implying that gene expression of HPGD might be regulated by negative feedback. Therefore, the increased HPGD protein in our study may also interfere syncytiotrophoblast cells of proliferation, migration, invasion, angiogenesis, and apoptosis. This finding is worthy of further investigation.

In addition, ERO1A, upregulated in our study, was strongly induced by hypoxia, involving in oxidative endoplasmic reticulum protein folding and inhibition of VEGF-driven angiogenesis [47]. Although the function of inhibiting VEGF-driven angiogenesis is similar to that of FLT1, the relationship between ERO1A and onset of PE remains unknown. More studies are required to investigate whether ERO1A is involved in the pathogenesis of PE and can be used as a potential biomarker.

Subsequently, we selected two proteins, FLT1, involved in the key processes as mentioned above, and PABPC4, verified by immunoblotting analysis. The expression pattern of these proteins was consistent with the proteomics results. Finally, bioinformatic analysis revealed that the differentially expressed proteins mainly enriched to apoptosis function and transcriptional misregulation in cancer pathways. These results implied that the regulation mechanism of syncytiotrophoblast on the invasion and migration of endometrium may be similar to that of tumour cells. Patients with GDM are more likely to have misregulation of transcription, angiogenic disorder, and oxidative stress, which lead to insufficient infiltration of syncytiotrophoblast cells and dysfunction of apoptosis, subsequently leading to the onset of severe early-onset PE. Further research and verification are needed to explore the possible.

Limitation of our study was that the sample size was small, for it is a little difficult to recruit more patients with strict inclusion criteria. A large number of experimental samples from different hospitals will be required to verify and obtain further arguments in future study. Moreover, proteomics studies of placental tissue are not the only way to confirm our findings, protein-related studies of fetal appendages such as blood, urine, and amniotic fluid are also feasible. Therefore, we hope that there will be more research on preeclampsia in the future.

## Conclusion

In this study, we performed TMT proteomic analysis on syncytiotrophoblast to compare the differentially expressed proteins. Twenty-three protein expressions were identified as significant differences between patients with GDM and patients with GDM with subsequently developed PE. Some of these differentially expressed proteins have been confirmed to be involved in the development of PE. Bioinformatic results indicated that the onset of PE form GDM patients also is a multifactorial disorder, involving in apoptosis, transcriptional misregulation, oxidative stress, cell infiltration and migration, angiogenesis, etc. Our results show that the inadequacy of endometrium infiltration, dysfunction of apoptosis, angiogenic disorder, and oxidative stress in syncytiotrophoblast cells more likely occur in GDM patients. These processes may be the potential mechanisms leading to GDM patients to secondarily developed severe early-onset PE. Thus, these proteins can also be considered as candidate biomarkers for predicting the onset of PE from women with GDM or as an intervention target for preventing GDM from developing into PE.

## Abbreviations

BMI: Body mass index
DBP: Diastolic blood pressure
EVT: Extravillous trophoblast cells
FABP4: Fatty acid-binding protein 4
FASP: Filter-aided sample preparation
FLT1: Vascular endothelial growth factor receptor 1
GAPDH: Glyceraldehyde-3-phosphate dehydrogenase
GDM: Gestational diabetes mellitus
GO: Gene ontology
HCD: Higher-energy collisional dissociation
HPGD: 15-hydroxyprostaglandin dehydrogenase
IGFBPs: Insulin-like growth factor binding proteins
IGFs: Insulin-like growth factors
KEGG: Kyoto encyclopedia of genes and genomes
LC-MS/MS: Liquid chromatography–mass spectrometry/ mass spectrometry
NF-κB: Nuclear factor kappaB
OGTT: Oral glucose tolerance test
PABPC4: Polyadenylate-binding protein 4
PE: Preeclampsia
PGE2: Prostaglandin E2
PPI: Protein–protein interaction
SBP: Systolic blood pressure
TMT: Tandem mass tag
TNF-α: Tumour necrosis factor alpha
VEGF: Vascular endothelial growth factor
